# Antigen-scaffolds loaded with hyper-stable neoleukin-2/15 expand antigen-specific T cells with a favorable phenotype for adoptive cell therapy

**DOI:** 10.3389/fimmu.2026.1804678

**Published:** 2026-06-11

**Authors:** Maria Ormhøj, Kamilla Kjærgaard Munk, Siri Tvingsholm, Keerthana Ramanathan, Amalie Kai Bentzen, Georgios Kladis, Gitte Nygaard Aasbjerg, Grigorii Nos, Tripti Tamhane, Hólmfridur Rósa Halldórsdóttir, Søren Nyboe Jakobsen, Marcus Svensson Frej, Kristoffer Haurum Johansen, Mohammad Kadivar, Sine Reker Hadrup

**Affiliations:** 1 Experimental and Translational Immunology, Department for Health Technology, Technical University of Denmark, Kongens Lyngby, Denmark; 2 Biotechnology, Department of Green Technology, University of Southern Denmark, Odense, Denmark

**Keywords:** adoptive cell therapy, artificial antigen-presenting scaffolds, neoleukin-2/15, T-cell expansion, T-cells

## Abstract

**Introduction:**

Adoptive cell therapy (ACT) has shown promising results in cancer treatment, however, achieving effective ex vivo expansion of potent, functionally active, and cytotoxic T cells remains challenging. To address this challenge, we recently developed artificial antigen-presenting scaffolds (Ag-scaffolds) capable of expanding antigen-specific T cells with phenotypes favorable for ACT. Here, we compared the established technology using IL2/IL21-loaded Ag-scaffolds (Ag-IL2/21) with scaffolds incorporating Neoleukin-2/15 (Ag-Neo2/15), an engineered cytokine that selectively signals via IL-2Rβ/γ complexes to enhance CD8^+^ T cell proliferation while limiting regulatory T cell expansion.

**Methods:**

Antigen-specific T cells were expanded ex vivo using Ag-IL2/21 or Ag-Neo2/15 scaffolds. Expansion efficiency, cytokine production, and cytotoxic activity were assessed. Functional and transcriptional states were profiled using single-cell sequencing, including cytotoxic and dysfunction gene signature scoring. T cell receptor (TCR) sequencing was performed to evaluate clonal expansion.

**Results:**

Ag-Neo2/15 scaffolds supported robust expansion of antigen-specific CD8^+^ T cells at levels comparable to Ag-IL2/21 scaffolds. Ag-Neo2/15-expanded CD8^+^ T cells exhibited increased effector cytokine production and durable cytotoxicity against tumor cells. Single-cell transcriptomic analysis revealed higher cytotoxic gene signature scores, reduced dysfunctional scores, and increased expression of genes linked to increased proliferation in Ag-Neo2/15-expanded T cells. TCR sequencing demonstrated preferential expansion of CD8^+^ T cell clones enriched for transcriptional features associated with antigen-experienced effector states and sustained effector function.

**Discussion:**

Ag-Neo2/15 scaffolds enhance the quality of *ex vivo*-expanded antigen-specific T cells by promoting a cytotoxic, less dysfunctional, and highly proliferative phenotype. These findings identify Ag-Neo2/15 as a promising improvement for ACT manufacturing, optimizing the balance between expansion capacity and durable effector function, with potential implications for improving cancer immunotherapy outcomes.

## Introduction

Adoptive cell therapy (ACT) relies on the infusion of *ex vivo* expanded T cells into cancer patients to generate a T cell-mediated antitumor response. ACT strategies can be broadly categorized into two approaches: I) T cells obtained from the patient’s blood, genetically engineered to express a tumor-specific T cell receptor (TCR) or a chimeric antigen receptor (CAR), and II) naturally occurring tumor-infiltrating lymphocytes (TILs) from surgically removed tumor samples. In recent years, CAR- or TCR-engineered T cells and TIL-based therapies have demonstrated remarkable clinical responses across multiple cancer settings. However, a common challenge across these strategies is the reliable generation of antigen-specific, highly functional, and persistent T cells that perform effectively *in vivo* (1–10). To overcome these challenges, we recently reported the development of artificial antigen-presenting scaffolds (Ag-scaffolds) for specific *ex vivo* expansion of circulating tumor-specific T cells from peripheral blood (11). Ag-scaffolds are composed of a dextran-polysaccharide backbone containing multiple streptavidin molecules, enabling site-specific conjugation of biotinylated proteins such as peptide-MHC (pMHC), cytokines, and co-stimulatory molecules. Using Ag-scaffolds decorated with pMHCs, IL-2, and IL-21, we were able to generate polyclonal T-cell products enriched for antigen-specific T cells with high self-renewal capacity, low exhaustion, and enhanced cytotoxicity (11).

Recently, *de novo*-designed Neoleukin-2/15 (Neo2/15) emerged as a hyper stable protein mimicking the functional properties of IL-2 and IL-15 (12). Neo2/15 specifically binds to the shared IL-2Rβ/γ receptor subunits (CD122/CD132), involved in T-cell activation while excluding the IL-2Rα receptor (CD25), thus reducing the activation of regulatory T cells (Tregs). In pre-clinical models, Neo2/15 treatment induced strong proliferation of CD8^+^ T cells with limited expansion of Tregs, leading to overall improvements in anti-tumor efficacy and survival (12). In this study, we assessed the efficacy of Neo2/15-loaded Ag-scaffolds (Ag-Neo2/15 scaffolds) in expanding antigen-specific T cells from peripheral blood mononuclear cells (PBMCs) of healthy donors using MHCs presenting viral peptides. We examined the effects of these scaffolds on T cell expansion kinetics, molecular characteristics, and functional properties, aiming to demonstrate that this technology can enable more effective T cell expansions.

## Results

### Antigen-presenting scaffolds loaded with Neo2/15 expand functional T cells

**Figure 1:**
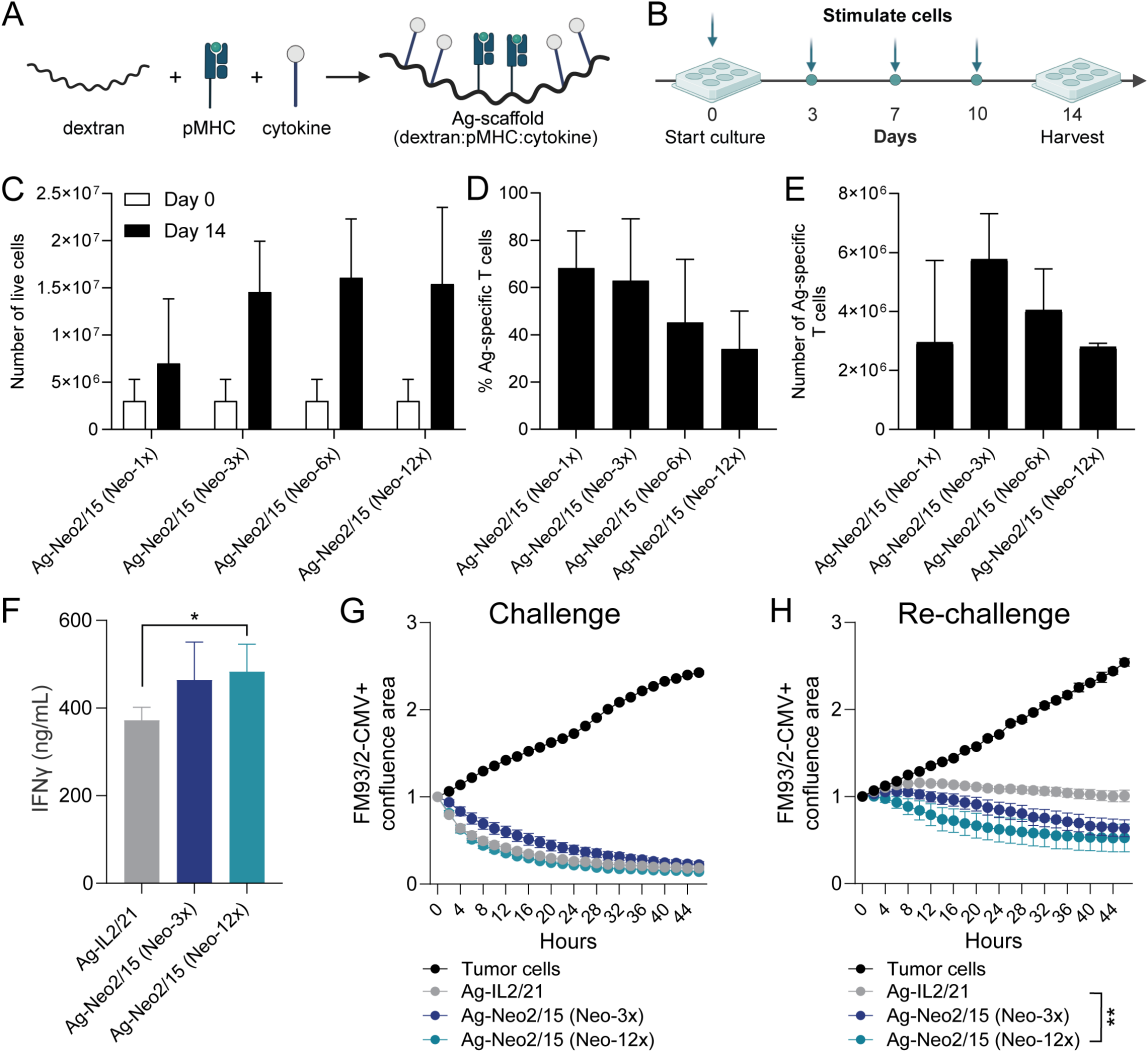
Neo2/15 induces strong proliferation and expansion of antigen-specific T cells. **(A)** Schematic representation of artificial antigen-presenting Ag-scaffolds **(B)** The experimental expansion setup. We used donors with viral responses to either the Influenza NP ILR-peptide bound to HLA-A*03:01 or the CMV pp65 TPR-peptide bound to HLA-B*07:02 with each graph representing data from 3 different donors. **(C)** Number of live cells days after expansion with Ag-Neo2/15 scaffolds. **(D)** Percent of antigen-specific T cells and **(E)** number of antigen-specific (Ag-specific) T cells in culture after 10 days of expansion. **(F)** The level of IFNg measured by ELISA in cell culture supernatant of Ag-scaffold expanded T cells co-cultivated with target cells. **(G)** The cytotoxicity of Ag-scaffold expanded T cells over a 44h co-culture period with target cells measured by Incucyte. **(H)** The cytotoxicity of Ag-scaffold expanded T cells over a 44h co-culture period when re-challenged with target cells measured by Incucyte. **(A, B)** were created in BioRender. Hadrup, S. (2026) https://BioRender.com/b0twcgp and https://BioRender.com/2ncb9zo. The statistical test shown between the different expansion strategies were compared using a one-way ANOVA test **(D–F)**, and 1-way ANOVA test test at time-point 46h **(G, H)**, where non-significant p-values are not shown, while p ≤ 0.05 and p < 0.01 are indicated by *, **, respectively.

To ensure site-specific conjugation of Neo2/15 to the dextran backbone, we designed a recombinant version of Neo2/15 carrying a C-terminal avitag (Neo2/15-avi). To test the functional activity of Neo2/15-avi, we used CFSE dilution to determine CD8^+^ T-cell proliferation. The activity of Neo2/15-avi was compared to recombinant IL-2. As the potency of Neo2/15-avi was unknown, we used molar concentration of Neo2/15-avi corresponding to 10, 100, and 1000 units of IL-2 for stimulation. Non-stimulated T cells were used as baseline, and T cells stimulated with anti-CD3/anti-CD28-coated Dynabeads and 20U/mL IL-2 as positive control. Eight days after stimulation, CFSE dilution was assessed by flow cytometry. Stimulation with 1000 U/mL of Neo2/15-avi or IL-2, respectively, resulted in similar levels of proliferation with more than 90% of CD8^+^ T cells having divided more than once (**Supplementary Figure 1**). Lower concentrations of IL-2 and Neo2/15-avi also resulted in a similar number of cell divisions (**Supplementary Figure 1**). Based on this, we concluded that Neo2/15-avi was fully functional and could be further assessed for expansion of antigen-specific T cells using the Ag-scaffold technology (**Figure 1A**). Ag-scaffolds are designed to ensure pMHC-directed expansion of antigen-specific T cells, while avoiding unspecific expansion of bystander T cells. To find the optimal molar ratio between dextran, pMHC, and Neo2/15-avi (dextran:pMHC: Neo2/15-avi), we assembled Ag-scaffolds with a constant pMHC amount, determined by the HLA type, and varying amounts of Neo2/15-avi relative to dextran. The resulting Ag-Neo2/15 scaffolds, had increasing molar ratio of Neo2/15 bound to the Ag-scaffold, here denoted Ag-Neo2/15 (Neo-1x), Ag-Neo2/15 (Neo-3x), Ag-Neo2/15 (Neo-6x), and Ag-Neo2/15 (Neo-12x). We used donors with viral responses to either the Influenza NP ILR-peptide bound to HLA-A*03:01 or the CMV pp65 TPR-peptide bound to HLA-B*07:02. These scaffolds were supplemented to cell cultures every three to four days during a 14-day expansion period (**Figure 1B**). Expansion with Ag-Neo2/15 scaffolds with higher Neo2/15-avi ratio resulted in significantly higher total numbers of cells after 14 days of culture (**Figure 1C**). The robust expansion and high viability observed across scaffold conditions argue against substantial fratricide occurring during culture. On day 14 of expansion, we performed tetramer staining to determine the percentage of antigen-specific T cells in the cultures (**Figure 1D**). When determining the total yield of antigen-specific T cells in the cultures we found the highest number of cells in cultures expanded with the Ag-Neo2/15 (Neo-3x) scaffold (**Figure 1E**). Next, we addressed the functionality of the expanded cells using flow cytometric analysis of intracellular cytokine staining. We observed a tight correlation between the overall frequency of triple-positive T cells (TNFα+/IFNγ+/CD107a+) and the percentage of antigen-specific T cells after co-culture with antigen-positive target cells (**Supplementary Figures 2A, B**). We observed lower level of PD-1 expression on CD8^+^ T cells expanded with higher number of loaded Neo2/15 molecules on the scaffold, while all the expanded T cells consistently expressed TIM3 and LAG3 across scaffold condition (**Supplementary Figure 2C**).

Next, we investigated the functional capacity of expanded T cells, in terms of killing target cells and production of the effector cytokine IFNγ. In this setup, we compared Ag-Neo2/15 scaffolds to IL-2/IL-21-loaded Ag-scaffolds with dextran:pMHC: IL-2:IL-21 ratio of 1:pMHC:6:6 (Ag-IL2/21 scaffolds) described by Tvingsholm et al. (10). We here selected Ag-Neo2/15 (Neo-3x) scaffolds because it demonstrated the high T cell expansion while maintaining a favorable functional profile, and Ag-Neo2/15 (Neo-12x) scaffolds to more closely resemble the cytokine quantity on the Ag-IL2/21 scaffolds. First, we measured the IFNγ release from expanded T cells after 24h of co-incubation with target cells. T cells expanded with Ag-Neo2/15 scaffolds produced more IFNγ as compared to T cells expanded with Ag-IL2/21 scaffolds (**Figure 1F**). The increased production of IFNγ was accompanied by superior cytotoxic functionality of T cells expanded with Ag-Neo2/15 scaffolds, enabling them to effectively kill target cells (**Figures 1G, H**; **Supplementary Figure 3**). Notably, this killing capacity was even more pronounced during the rechallenge compared to the initial 46 hours of co-culturing with target cells (**Figure 1H**; **Supplementary Figure 3**).

### Single-cell analysis of antigen-specific T cells expanded with Ag-Neo2/15 scaffolds

**Figure 2:**
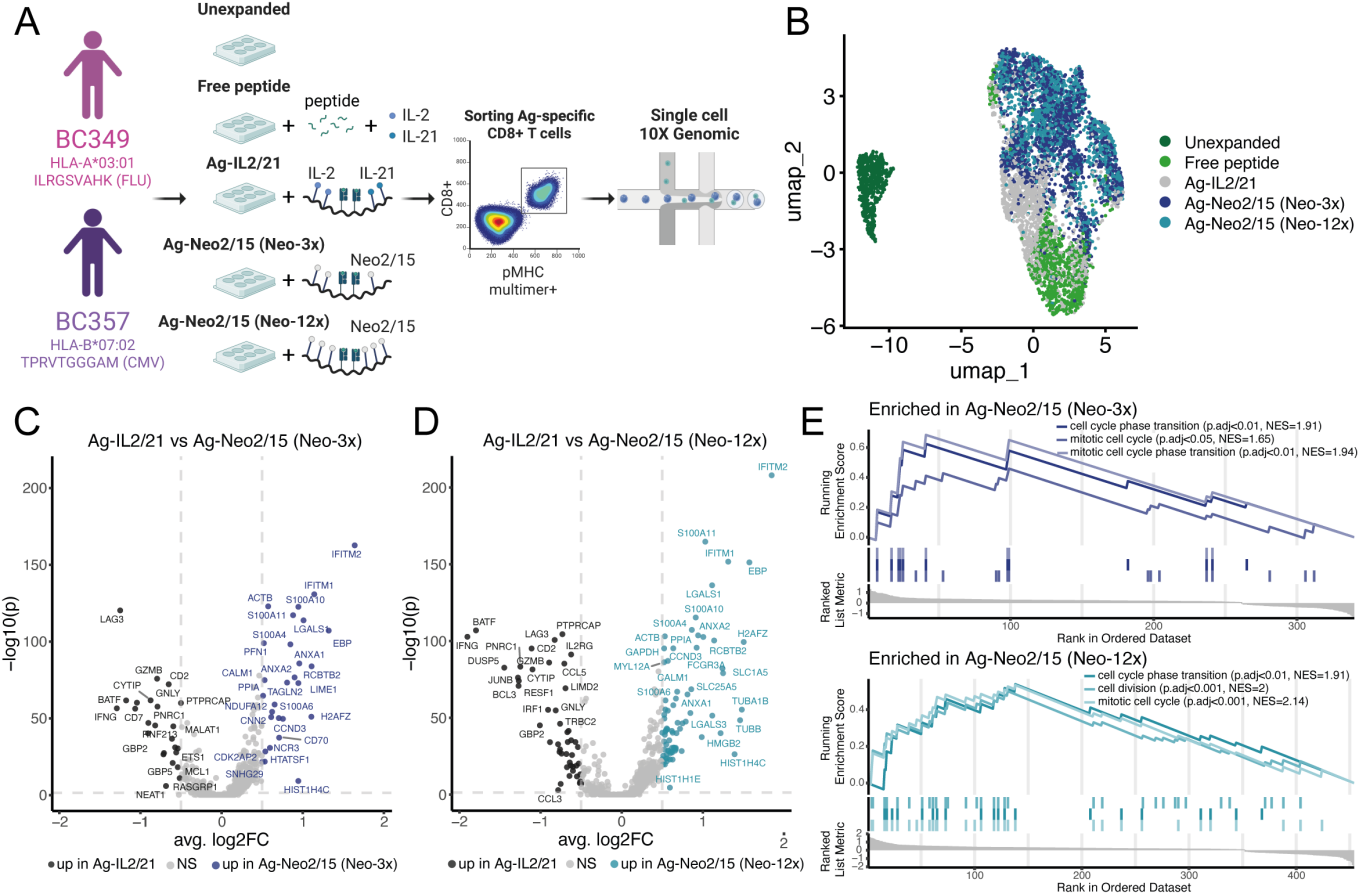
Antigen-specific CD8+ T cell expanded with Neo-2/15 exhibit a distinct transcriptomic profile. **(A)** Experimental workflow for the single-cell analysis of CD8+ and multimer+ T cells from human PBMCs under different *in vitro* expansion strategies. Using T cells from HLA-A*03:01 Influenza NP IRL-specific and HLA-B*07:02 CMV pp65 TPR-specific T cells from two healthy donors. **(B)** UMAP projection of the scRNA-seq data colored based on the five different expansion strategies. **(C)** Volcano plot showing key differentially expressed genes between the Ag-IL2/21 and Ag-Neo2/15 (Neo-3x) and **(D)** between the Ag-IL2/21 and Ag-Neo2/15 (Neo-12x). **(E)** Selected biological pathways determined by the gene set enrichment analysis when comparing Ag-IL2/21 and Ag-Neo2/15 (Neo-3x) (top panel) and when comparing Ag-IL2/21 and Ag-Neo2/15 (Neo-12x) (bottom panel). **(B)** was created in BioRender. Hadrup, S. (2025) https://BioRender.com/50fizay. Data is shown for T cells exapnded from 2 donors (n=2).

After observing the enhanced killing capacity of CD8+ T cells expanded with Ag-Neo2/15 scaffolds, we performed a single-cell analysis using antigen-specific T cells from two healthy donors to further investigate differences between cells expanded with Ag-Neo2/15 scaffolds and those expanded using conventional strategies. In this analysis, we examined the transcriptome, surface proteome, and T-cell clonality of unexpanded T cells, as well as T cells expanded using free peptide, IL-2 and IL-21, Ag-IL2/21 scaffolds, and two different Ag-Neo2/15 scaffolds (Neo-3x and Neo-12x) (**Figure 2A**). The donor-specific T cells were cultured for two weeks and stimulated twice weekly until harvest (**Supplementary Figure 4**). At harvest, CD8+ pMHC tetramer+ T cells (i.e., antigen-specific CD8+ T cells) were sorted based on donor-specific viral responses to either the Influenza NP ILR-peptide bound to HLA-A*03:01 or the CMV pp65 TPR-peptide bound to HLA-B*07:02 and analyzed by single-cell transcriptomics (**Figure 2A**, **Supplementary Figure 4**).

UMAP analysis of the transcriptome revealed distinct clusters of T cells under the various expansion conditions (**Figure 2B**). Unstimulated cells formed a separate cluster (on the left), suggesting a unique transcriptional profile, while cells expanded with free peptide and cytokines, Ag-IL2/21 scaffolds, and the two Ag-Neo2/15 scaffolds (Neo-3x and Neo-12x) clustered closely, indicating less transcriptional differences. The same trend was observed when evaluating the differential expressed genes within each expansion condition (**Supplementary Figure 5**). Donor concordance was assessed using donor-split UMAPs and Pearson correlation of mean gene expression per expansion condition, showing high agreement between donors (r = 0.91 - 0.98; **Supplementary Figure 6**), indicating minimal donor-driven effects.

We have previously demonstrated the superiority of the Ag-IL2/21 scaffold when compared to more traditional expansion methods like free peptides and cytokines (10); therefore, we chose the Ag-IL2/21 scaffold as our most advanced expansion method for comparison to Ag-scaffolds loaded with Neo2/15, to investigate how the different cytokines affect the final T-cell products. To investigate the difference between T cells expanded with Ag-IL2/21 scaffolds and cells expanded with Ag-Neo2/15 scaffolds, we performed a differential expression analysis (DEA) (**Figures 2C, D**; **Supplementary Tables 1**, **2**), followed by a gene set enrichment analysis (GSEA) (**Figure 2E**; **Supplementary Figures 7**, **8**). T cells expanded with Ag-Neo2/15 scaffolds showed higher expression of genes linked to cell division and mitotic cell cycle (TUBA1B, TUBB, CCND3, CDK2AP2), indicating that these are more proliferative than T cells expanded with Ag-IL2/21 scaffolds. Interestingly, this also resulted in a higher number of antigen-specific CD8^+^ T cells in cultures expanded with Ag-Neo2/15 scaffolds compared to Ag-IL2/21 scaffolds (**Supplementary Figure 4C**). Meanwhile, T cells expanded with Ag-IL2/21 have a higher expression of genes linked to T cell effector functionality (CCL5, IL2RG, IFNG, IL2RG, BATF, IRF1).

Similar trends are evident when examining surface markers derived from the single cell analyses (**Supplementary Figures 9**, **10**). Notably, CD8^+^ T cells expanded with Ag-Neo2/15 scaffolds displayed higher expression of the inducible costimulatory receptor ICOS compared to those expanded with Ag-IL2/21 scaffolds. ICOS is expressed on activated T cells and plays a role in T cell proliferation, cytokine production, and survival. However, CD8^+^ T cells expanded with Ag-IL2/21 scaffolds exhibited elevated levels of markers associated with activation (CD69, CD7, CD2 and CD27) and exhaustion (CD39) (**Supplementary Figures 9**, **10**).

Interestingly, the expression of homing molecules differed between cells expanded with either Ag-IL2/21 or Ag-Neo2/15 scaffolds. CD8^+^ T cells expanded with Ag-Neo2/15 scaffolds showed higher expression of the intestinal homing molecule, alpha4-beta7 integrins (Integrin beta7 and CD49d); whereas those expanded with Ag-IL2/21 exhibited increased expression of more generic homing molecules, including CD103 (alphaE-beta7 integrin homing to epithelial tissues) and CD11a (forming LFA-1 together with CD18). This suggests that the different scaffolds can affect the expression of homing molecules on the expanded T cells, guiding them to specific tissue types.

Overall, our single-cell analysis revealed that T cells expanded with Ag-Neo2/15 scaffolds exhibited distinct transcriptional profiles and higher proliferation rates compared to those expanded with Ag-IL2/21 scaffolds. Ag-Neo2/15-expanded T cells showed increased expression of genes linked to cell division and higher levels of the inducible costimulatory receptor ICOS. In contrast, Ag-IL2/21-expanded T cells had higher expression of activation and differentiation genes, along with elevated levels of activation and exhaustion surface markers.

### T cells expanded with Ag-Neo2/15 scaffolds have favorable functional phenotype characteristics

**Figure 3:**
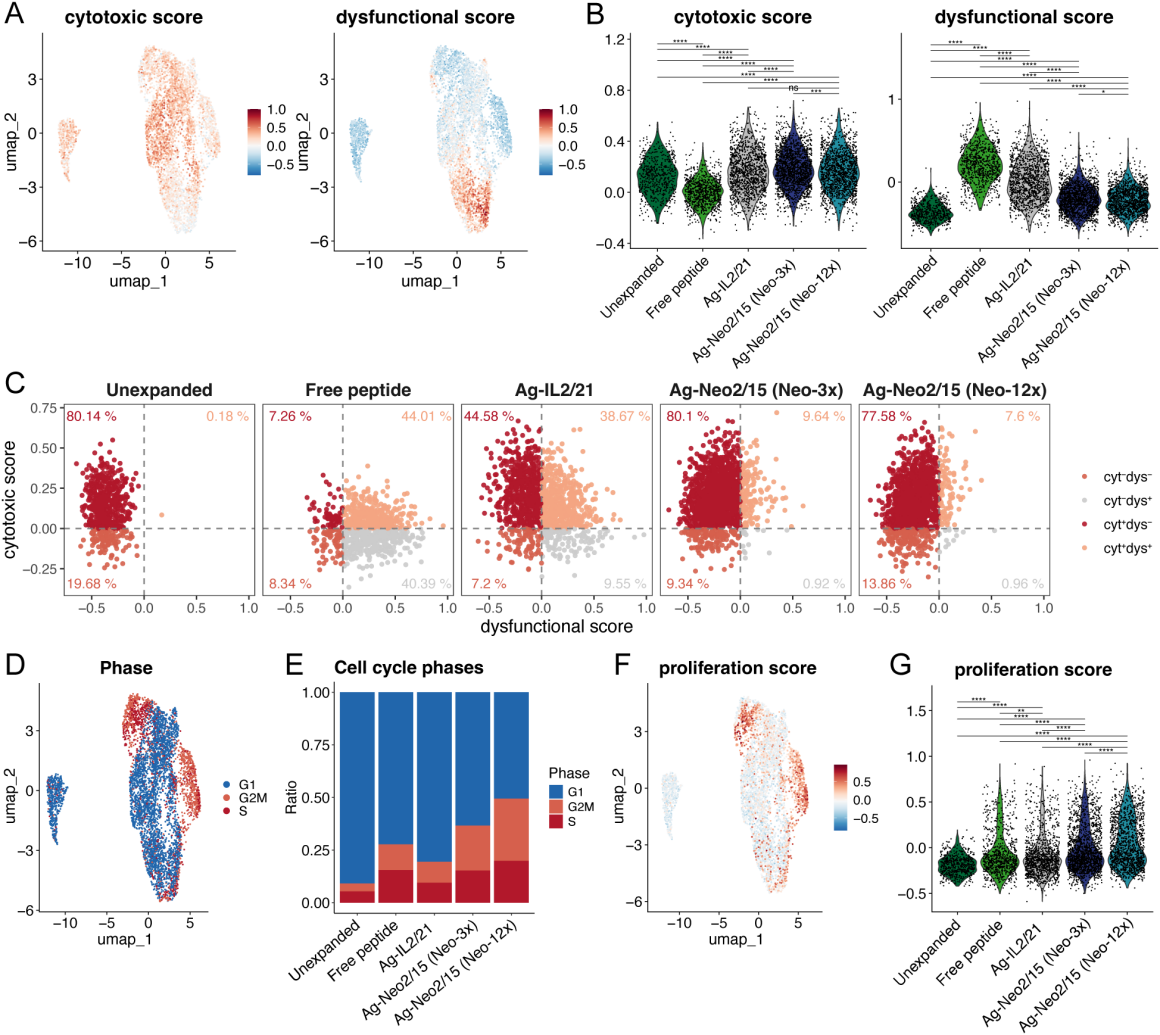
T cells expanded with NeoIL2/15 have a more favorable phenotype and shows tumor killing. **(A)** UMAP shows the cytotoxic and dysfunctional score for each cell. **(B)** Violin plots showing the expression level of the cytotoxic and dysfunctional score for each cell within each of the five expansion strategies. **(C)** Quadrant scatter plots showing the relationship between the cytotoxic and dysfunctional score for each cell for each expansion strategy. Each scatter plot is divided into four quadrants. The four quadrants represent: cyt^+^dys^+^ (both scores > 0), cyt^-^dys^-^ (both scores < 0), cyt^+^dys^-^ (cytotoxic > 0, dysfunctional < 0), and cyt^-^dys^+^ (cytotoxic < 0, dysfunctional > 0). **(D)** UMAP showing cell cycle phase G1, G2M and S for each cell. **(E)** Ratio of the three cell cycle phases for each expansion strategy. **(F)** UMAP showing proliferation score for each cell. **(G)** Violin plots showing the expression level of the proliferation score for each expansion strategy. The statistical test shown between the five different expansion strategies were compared using an unpaired, non-parametric Wilcoxon test of the mean expression, where p values > 0.05 were considered statistically non-significant and represented as ns, whereas p ≤ 0.05, p < 0.01, p < 0.001 and p < 0.0001 is represented as *, **, *** and ****, respectively. Data is shown for T cells expanded from 2 donors (n=2).

To assess the functional state of the different expansion conditions, we used the top 30 cytotoxicity (cyt) and dysfunctionality (dys) gene signatures, described by Li et al. (13), to calculate a combined score for each gene set. The resulting scores for each cell were visualized using UMAPs (**Figure 3A**), and the differences between these scores for each expansion strategy were quantified (**Figure 3B**).

T cells expanded with Ag-Neo2/15 scaffolds showed significantly higher cytotoxicity scores compared to both unstimulated and free peptide-expanded cells, whereas T cells expanded with Ag-IL2/21 scaffolds had more comparable cytotoxicity scores. This suggests that cells expanded with Ag-Neo2/15 scaffolds induce a potent cytotoxic T-cell response similar to that seen with Ag-IL2/21 scaffolds (**Figure 3B**).

Furthermore, Ag-Neo2/15-expanded T cells exhibited significantly lower dysfunctionality scores compared to those expanded with either free peptides or Ag-IL2/21 scaffolds (**Figure 3B**). As expected, unexpanded cells had the lowest dysfunctionality scores, as these cells have not been expanded and, therefore, have not undergone repeated activation or stimulation that could lead to exhaustion or dysfunction.

To further investigate the relationship between the cytotoxicity and dysfunctionality score for each cell, we generated a scatter plot for each of the different expansion conditions (**Figure 3C**). This plot underscores the effect that the different expansion conditions have on the T-cell phenotype. Notably, Ag-Neo2/15-expanded cells have a higher proportion of both cyt^+^dys^–^ phenotypes, with fewer cells in the cyt^–^dys^+^ quadrants. This indicates that the Ag-Neo2/15 scaffold expansion not only promotes enhanced cytotoxicity but also preserves T-cell functionality by reducing the expression of dysfunction-associated genes.

These results highlight that expansion with Ag-Neo2/15 scaffolds maintain a cytotoxic phenotype while having lower dysfunctionality scores, distinguishing it from other expansion conditions. The higher cytotoxicity scores observed in Ag-Neo2/15-expanded T cells, suggest that this Ag-scaffold may provide a powerful tool for enhancing T-cell-based immunotherapies. Additionally, the reduced dysfunctionality scores in Ag-Neo2/15-expanded T cells indicate that this approach helps maintain T-cell functionality, potentially improving the efficacy of adoptive T-cell therapies.

Based on the findings in **Figure 1**, which suggest that Ag-Neo2/15 scaffold expansion promotes a more proliferative state in T cells, we aimed to validate this observation using the single cell data. Looking into the cell cycle phase dynamics, we observe that most cells across all conditions remain in the G1 phase, where cells grow and prepare for DNA replication (**Figures 3D, E**). Smaller populations were observed in the S phase, where DNA replication occurs, and in the G2M phase, associated with cell division. However, the cell cycle phase distribution differed among the different expansion conditions (**Figure 3E**). Notably, Ag-Neo2/15-expanded cells had an increased proportion of cells in the S and G2M phases, suggesting that these cells are more proliferative. Furthermore, proliferation scores, calculated from the mean expression for a defined proliferation gene set, further confirmed this trend (**Figures 3F, G**). As seen on the UMAP and quantified in violin plots, cells expanded with Ag-Neo2/15 scaffolds consistently exhibit higher proliferation scores than those left unexpanded or expanded with free peptides or Ag-IL2/21 scaffolds.

Collectively, these results suggest that Ag-Neo2/15 scaffold expansion promotes a highly proliferative, cytotoxic T cell with reduced dysfunctionality and favorable functional phenotype capacity, supporting its potential application in adoptive cell therapies.

### Ag-Neo2/15 scaffolds expand T cell clones with favorable functional phenotype characteristics

**Figure 4:**
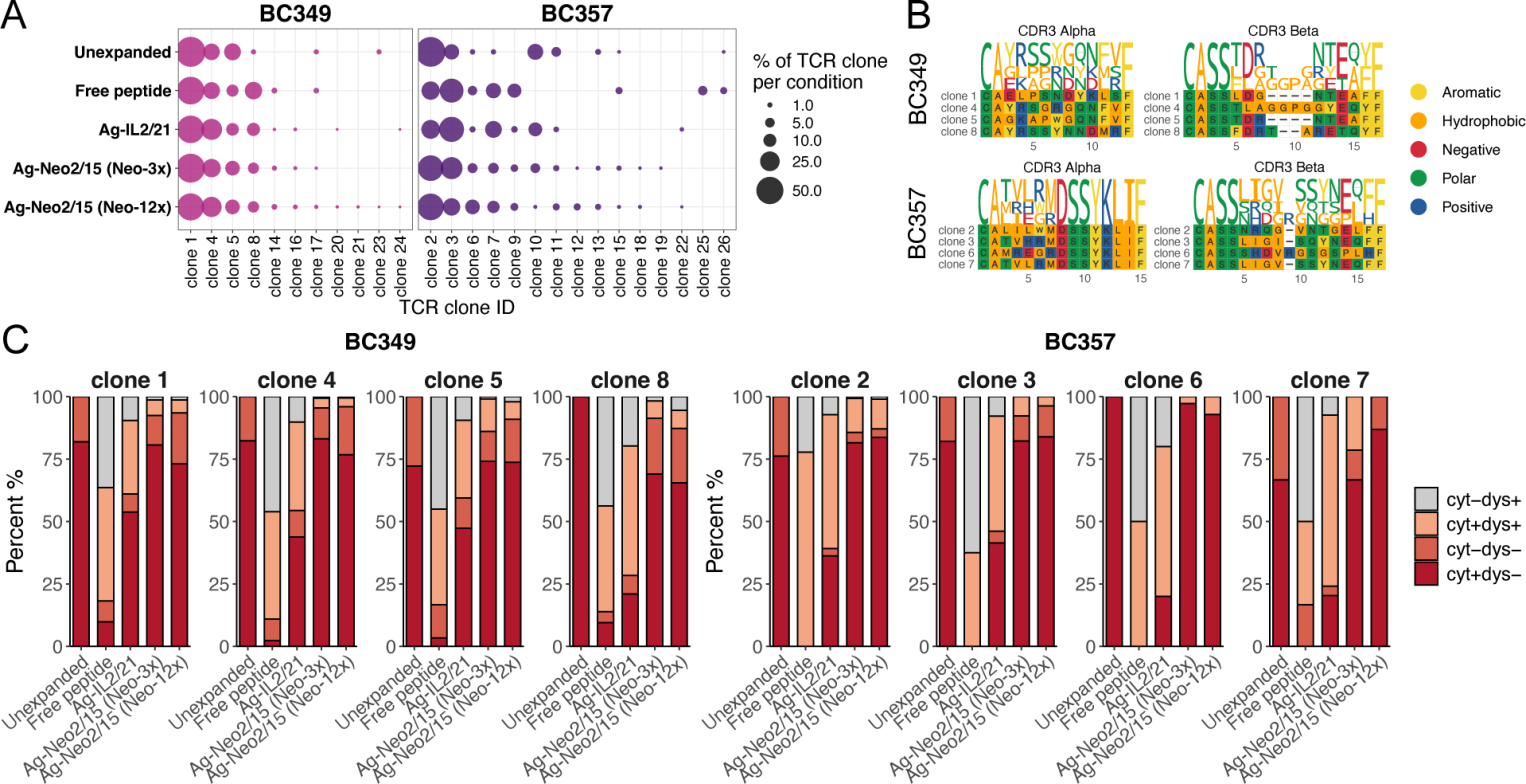
TCR diversity and clonal expansion. **(A)** TCR clonotype distribution of the Ag-specific T cells from the single cell experiment. Circle size corresponds to clonal frequency out of all TCR clones detected within each expansion strategy. **(B)** UMAPs of the top four TCR clonotypes for each of the two healthy donors, BC349 (left) and BC357 (right). **(C)** Distribution of the relationship between the cytotoxic and dysfunctional score for each of the five expansion strategies within the top four TCR clonotypes for each of the two donors. The four colors represent: cyt^+^dys^+^ (both scores > 0), cyt^-^dys^-^ (both scores < 0), cyt^+^dys^-^ (cytotoxic > 0, dysfunctional < 0), and cyt^-^dys^+^ (cytotoxic < 0, dysfunctional > 0). Data is shown for T cells exapnded from 2 donors (n=2).

To further elucidate differences between the T-cell products generated under the various expansion conditions, we analyzed the TCR clone distribution for each condition (**Figure 4A**). This analysis revealed that the expansion of TCR clones was largely similar across expansion conditions (**Figure 4A**). However, some variation was observed especially for BC357 indicating that the clonal expansion can be affected by the cytokine loaded onto the scaffolds. To investigate the similarity of these clones, we compared the CDR3 alpha and beta sequences for the top four T-cell clones per donor and showed that these sequences are quite distinct and differs in both length and minimal sequence similarity (**Figure 4B**, **Supplementary Table 3**). Even though the T cells from each donor only recognize a single peptide-MHC complex, these T cells maintain a diverse TCR repertoire, an observation that aligns with previous findings (14). Looking further into the phenotypic characteristics of the four most dominant TCR clones for each donor, we found that the top clones expanded with Ag-Neo2/15 scaffold had a higher proportion of cyt^+^dys^–^ (**Figure 4C**; **Supplementary Figure 11**). This indicates that both the phenotype and clonality of the clones are influenced by the different expansion conditions.

## Discussion

This study demonstrates the successful development and optimization of an antigen-specific T cell expansion platform utilizing Neo2/15, an engineered neoleukin IL-2/IL-15 chimera, conjugated to dextran-based antigen scaffolds. Our findings reveal significant advantages of this system for generating high-quality antigen-specific T cells for adoptive cell therapy applications.

The results show that Neo2/15 has the ability to stimulate CD8^+^ T-cell proliferation to comparable levels as recombinant IL-2 in our setting. This finding is noteworthy as Neo2/15 is engineered to reduce side effects commonly associated with IL-2 therapy, such as vascular leak syndrome (VLS), while maintaining potent immunostimulatory properties (15). The comparable proliferation rates observed in our study confirm that Neo2/15 can be a relevant alternative to IL-2 in T-cell expansion protocols. Building on this validation, we incorporated the recombinant Neo2/15 to previously established antigen scaffolds to expand antigen-specific CD8^+^ T cells. Importantly, CD8^+^ T cells expanded with the optimized Ag-Neo2/15 scaffolds maintained high functionality, as assessed by cytokine production (TNFα, IFNγ) and degranulation (CD107a) upon antigen re-stimulation.

Our findings demonstrate that T cells expanded with Ag-Neo2/15 scaffolds exhibit favorable functional phenotype characteristics compared to those expanded with Ag-IL2/21. Notably, Ag-Neo2/15-expanded T cells produced significantly higher levels of IFNγ upon co-incubation with target cells, indicating an effector response. The killing assay results revealed that these T cells maintained superior cytotoxicity against target cells, even upon rechallenge. This enhanced killing capacity underscores the potential of Ag-Neo2/15 scaffolds to generate highly effective T cells for adoptive cell therapy, capable of sustained activity in immunosuppressive environments. Previous studies have demonstrated the potential of Neo2/15 in preclinical models, suggesting that Neo2/15 can significantly enhance the cytotoxic capacity of T cells, making them more effective in targeting and eliminating tumor cells (12, 16).

To further investigate T cells expanded using Ag-Neo2/15 scaffolds and compare them with other expansion strategies, including our recently established Ag-scaffold technology Ag-IL2/21 (11), we performed single-cell analysis on CD8^+^ T cells. This setup included unexpanded T cells and T cells expanded with either free peptide and cytokine (a conventional expansion method) or Ag-scaffolds decorated with pMHC, IL-2, and IL-21, as described previously by our group (11). Our transcriptomic analysis revealed differences between expanded CD8^+^ T cells using Ag-Neo2/15 scaffolds and those expanded by other methods. Notably, CD8^+^ T cells expanded with Ag-Neo2/15 scaffolds at both stoichiometries (Neo-3x and Neo-12x) exhibited considerable overlap in their transcriptional profiles, suggesting that the concentration difference had minimal impact on the overall transcriptional state of these cells. Differential expression analysis followed by GSEA indicated that CD8^+^ T cells expanded with Ag-Neo2/15 scaffolds showed increased expression of genes linked to cell division and proliferation capacity. Furthermore, cell cycle analysis demonstrated that Ag-Neo2/15-expanded cells had an increased proportion of cells in the S and G2M phases, suggesting enhanced proliferative capacity. This aligns with recent studies highlighting the importance of promoting T-cell proliferation while maintaining effector functions in adoptive cell therapy (17, 18). The ability to generate large numbers of highly functional T cells is a key factor in the success of cellular immunotherapies. This gene signature suggests that Ag-Neo2/15 scaffold expansion more effectively promotes T cell proliferation. The ability to induce robust proliferation while maintaining functional capacity is a crucial factor in developing effective T-cell based therapies.

Our immunophenotyping analysis on the single cell platform suggests that different expansion conditions significantly influence the homing characteristics and potential functionality of CD8^+^ T cells. Ag-Neo2/15 scaffold expanded cells indicate a preference for intestinal homing (α4β7 integrins), while Ag-IL2/21 scaffold expanded cells express more general homing molecules (CD103, CD11a/LFA-1). This distinction could have significant implications for the tissue tropism and migratory behavior of these expanded CD8^+^ T cells. The Ag-Neo2/15 scaffold expanded cells might be more suited for targeting intestinal tissues, whereas the Ag-IL2/21 expanded cells could have broader tissue-homing capabilities, including epithelial tissues. Moreover, the higher expression of ICOS on Ag-Neo2/15 scaffold expanded cells indicates a more costimulatory phenotype, which could lead to enhanced T-cell activation and function. ICOS is the hallmark of CD8+ tissue-resident memory T cells and is induced by T-cell activation and promotes T-cell proliferation and survival (19).

Our single-cell RNA sequencing analysis also revealed that T cells expanded with Ag-Neo2/15 scaffolds exhibited more favorable characteristics compared to the Ag-IL2/21 scaffold and free peptide expansion methods. Cells expanded with Ag-Neo2/15 scaffolds showed significantly higher cytotoxic scores and lower dysfunction scores, indicating a potent effector phenotype with reduced exhaustion markers. This aligns with our killing assay results, which demonstrated that T cells expanded with Ag-Neo2/15 scaffolds show enhanced functional killing capacity compared to those expanded with Ag-IL2/21 scaffolds. This finding is particularly important in the context of adoptive T-cell therapy, where maintaining T-cell functionality is crucial for therapeutic efficacy (17).

Our analysis of TCR clonality revealed that using the Ag-Neo2/15 scaffold expansion method may influence the distribution of dominant TCR clones in a donor-dependent manner. Moreover, the functional characteristics of expanded clones were markedly improved when expanded with Ag-Neo2/15 scaffolds, showing a higher proportion of cytotoxic and functional T cells. This suggests that our expansion method using Ag-Neo2/15 scaffolds promotes expansion of the T clones with enhanced functional quality, a crucial factor in maintaining diverse antigen recognition capabilities. The observation of distinct CDR3 sequences among the top clones, despite their identical antigen specificity, aligns with previous findings on TCR diversity in antigen-specific responses (14). This diversity likely contributes to a more robust immune response by allowing for varied recognition strategies of the same antigen, potentially enhancing the overall efficacy of the T cell product (17).

Our findings have significant implications for the field of adoptive T-cell therapy. The Ag-Neo2/15 scaffold expansion method offers a promising approach for generating large numbers of functional antigen-specific T cells. This approach has the potential to enhance the production of high-quality T cells for adoptive immunotherapy applications, potentially leading to improved clinical outcomes in cancer and infectious disease treatments.

## Methods

### Cell lines and healthy donor T cells

The HLA-A*0201-positive FM93/2 (ESTDAB-007) melanoma tumor cell lines were purchased from the European Searchable Tumor Cell Line Database (ESTDAB) and cultured as per supplier recommendation in RPMI media containing 10% FBS and 1% pen-strep. To establish a CMV pp65 NLVPMVATV positive cell line, FM93/2 cells were lentivirally transduced with a bicistronic vector encoding click beatle green luciferase (CBG), mCherry fluorescent reporter, mouse mammary tumor virus (MMTV) gp70 signal peptide followed (20) by the NLVPMVATV peptide sequence (FM93/2-CMV+). FM93/2-CMV+ cells were single cells sorted to establish a clonal cell line. As a negative control, we generated an FM93/2 cell line solely expressing CBG-Luc and mCherry (FM93/2-CMV-). Peripheral blood mononuclear cells (PBMCs) were obtained from healthy donor buffy coats collected after informed consent and in accordance with the declaration of Helsinki from the central blood bank at Copenhagen university hospital (Rigshospitalet). Prior to expansion, HLA typing was performed, and T-cell reactivity was assessed against a panel of viral epitopes using pMHC tetramers (11).

### Production of avitagged, biotinylated Neo2/15

The DNA sequence encoding Neo2/15 (12) was synthesized and cloned into the pET-30 plasmid between an N-terminal His-tag and C-terminal avitag for protein expression in *E.coli* (Neo2/15-avi) (Genscript). The E. coli strain BL21 (DE3) was co-transformed with plasmid and BirA for recombinant protein production. Briefly, a single colony was grown in TB media at 37 °C until an OD600 value of 1.2 was reached followed by isopropyl β-D-1-thiogalactopyranoside (IPTG) induction at 15 °C for 16h. E. coli was pelleted by centrifugation and the supernatant collected for purification. Recombinant Neo2/15-avitagged protein was purified by one-step purification using a Ni-NTA column and sterilized by a 0.22μm filter before being stored in aliquots. The concentration was determined by BCATM protein assay with BSA as standard. The protein purity and molecular weight were determined by standard SDS PAGE along with Western blot confirmation. Neo2/15-avi was biotinylated using BirA biotin-protein ligase standard reaction kit (Avidity, LLC Aurora, Colorado) and purified with size exclusion chromatography using HPLC (Waters Corporation, USA). The protein concentration was measured using the Bradford assay, with BSA as the standard. Biotinylation efficiency was assessed via a gel-shift assay: briefly, biotinylated Neo2/15 forms multimers upon coupling with streptavidin, which were analyzed using standard SDS-PAGE.

### pMHC, tetramer and Ag-scaffold production

Relevant peptides were purchased from pepscan (Pepscan Presto BV), dissolved to 10 mM in DMSO and stored at -20 °C until use. Recombinant Human Leukocyte Antigen (HLA) heavy chains and human β2 microglobulin light chain were produced in *E.coli*. We used a functionally empty disulfide-stabilized peptide-receptive HLA-A*02:01 variant (21), which can be loaded with peptide of interest during a 15-minute incubation at room temperature. For HLA-A*01:01 and HLA-B*07:02, the heavy and light chains were refolded with ultraviolet (UV)-sensitive ligands and purified as previously described (11) and pMHC complexes were generated by UV-mediated exchange with relevant peptides.

Tetramers were generated by conjugating pMHC monomers and fluorochrome labeled streptavidin. Blocking of free streptavidin sites was done with 25 uM D-Biotin (Avidity, BIO-200).

Ag-scaffolds were assembled, using a streptavidin-conjugated dextran backbone (500 kDa) (Fina Biosolutions) mixed with biotinylated relevant pMHC molecules and Neo2/15-avi or avitagged IL-2 (Acro IL-2H82F3) and IL-21 (Acro IL-21-H82F7). Potential free streptavidin sites on the dextran backbones were blocked with D-biotin. To remove unbound molecules, Ag-scaffolds were filtered through 100kDA columns (Vivaspin6, Sartorius). Ag-scaffolds were stored at −80 °C in a 10x freezing solution containing 5% Bovine Serum Albumin (BSA) and 50% Glycerol.

The dextran:pMHC ratio was experimentally determined for each HLA. The ratio for Ag-scaffold with HLA-A*02 was 1:36 or 1:24 depending on the batch, HLA-B*07 was 1:24 and HLA-A*03 was 1:24.

### Expansion of antigen-specific T cells using Ag-scaffolds

Specific T cells with pre-defined viral responses were expanded from PBMCs over a 10 to 14-day period. PBMCs were cultured in low-attachment culture plates (2–3 mio./ml) in VIVO 15 media (Lonza, BE02-060Q) supplemented with 5% human serum (Sigma Aldrich Heat Inactivated H3667) and supplemented with Ag-scaffold (0.16 nM prefiltration) or specific peptide (5 µM), soluble IL-2 (40 U/mL, Peprotech 200–02) and soluble IL-21 (25 ng/mL, Peprotech 200–21) (free peptide condition). Ag-scaffold or free components were supplemented to the culture at initiation and twice weekly until harvest. Upon signs of over-confluence, cultures were transferred to larger plates and culture volumes while maintaining the Ag-scaffold concentration.

### T-cell cytotoxicity assay

The cytotoxic potential of Ag-scaffold expanded T cells was measured using the IncuCyte (Satorious). FM93/2-CMV+ cells or FM93/2-CMV- control cells were plated at a density of 5,000 cells/well and allowed to attach overnight. The following day, Ag-scaffold expanded T cells were added in an effector: target ratio of 1:1, and the plate was placed in the IncuCyte instrument. Data recording was set to 2-hour intervals over two days, and changes in impedance were reported as confluence areas. After the initial two-day measurement, the plate was removed from the Incucyte, cells were pelleted, and cell culture supernatant was collected for IFNγ ELISA. The pelleted cells were re-suspended in fresh medium and transferred to a new plate containing 5,000 FM93/2-CMV+ cells or FM93/2-CMV- control cells. Both FM93/2-CMV+ cells or FM93/2-CMV- control cells were plated 24h prior to rechallenge. The plate containing target and effector cells were placed in the IncuCyte and T-cell killing was followed for an additional two-day period.

### Intracellular cytokine staining

Expanded antigen-specific T cells were incubated with peptide-pulsed T2 target cells (E:T 1:1) for 4 hours in X-VIVO 15 media supplemented with αCD107a-PE antibody (BD 555801) and GolgiPlug (BD 550583). Co-incubation of antigen-specific T cells with non-pulsed T2 cells was used as negative control and co-incubation of antigen-specific T cells with non-pulsed T2 cells and leucocyte activating cocktail (LAC, BD, 550583) was used as positive control. Harvested Tcells were stained with LIVE/DEAD Fixable Near-IR cell marker (Invitrogen) for dead cell exclusion, CD3-FITC (BD 345764) and CD8-BV480 (BD 566121), fixed and permeabilized (Invitrogen 88882400) and stained with TNFα-PE-Cy7 (Biolegend 502930) and IFNγ-APC (BD 341117) and analyzed by flow cytometry.

### IFNγ ELISA

IFNγ in culture supernatants was monitored by ELISA. Briefly, wells were coated with (1µg/mL) anti-IFNγ capture antibody (BD 551221) prior to the addition of cell culture supernatant. The presence of IFNγ was detected using biotinylated 0,5µg/mL anti-IFNγ antibody (BD 554550). The reaction was visualized by a peroxidase-streptavidin conjugate (1:10,000; Merck 11089153001) and TMB (Kementec 4380A).

### 10xGenomics single-cell analysis platform

Cryopreserved PBMCs were thawed and stained with TotalSeq™-C Human Universal Cocktail V1.0 (Biolegend), TotalSeq-C anti-human Hashtag antibodies for each distinct sample (BioLegend), LIVE/DEAD Fixable Near-IR cell marker (Invitrogen), and flow cytometry antibodies in presence of Human TruStain FcX Fc Blocking reagent (Biolegend) to block Fc receptors. Cell surface protein expression was assessed using oligonucleotide-conjugated antibodies from the TotalSeq™-C Human Universal Cocktail (BioLegend) in combination with the 10x Genomics Feature Barcoding technology. These antibodies enable simultaneous quantification of surface protein expression alongside transcriptomic profiling by sequencing antibody-derived tags (ADTs). Cells were stained according to the manufacturer’s protocol, including Fc receptor blocking to minimize non-specific binding. Following sequencing, ADT counts were processed and normalized using the Seurat package, allowing integration of surface protein expression with gene expression data at the single-cell level. The flow cytometry antibody mix was composed of CD8-BV480 (BD 566121, clone RPA-T8) and lineage antibodies: CD4-FITC (BD 345768), CD14-FITC (BD 345784), CD19-FITC (BD 345776), CD40-FITC (Serotech MCA1590F), CD16-FITC (BD 335035). The stained cells were sorted as single, live, CD8+ tetramer-positive cells on a FACS Melody (BD) cell sorter for subsequent processing on the 10x Genomics platform. We utilize the 10xGenomics 5′ v2 chemistry that allows us to use the immunophenotyping antibodies. Downstream processing of mRNA and DNA barcodes is performed according to the manufacturer’s instructions (Chromium Next GEM Single Cell 5’ Reagent Kits v2 [Dual Index], with the Feature Barcode technology for Cell Surface Protein & Immune Receptor Mapping) (10x Genomics, USA). The TCR, barcode (including immunophenotyping antibodies, and hashtag antibodies), and gene expression libraries were sequenced by Novogene on a NovaSeq running a 150 paired-end program.

### Single-cell data processing

The raw FASTQ files were processed using the CellRanger multi pipeline (10x Genomics, V.6.1.1), as this enables simultaneous profiling of the T-cell V(D)J repertoire, immunophenotyping antibodies (TotalSeq™-C), sample hashing, and gene expression. Both the V(D)J and gene expression were aligned to the GRCh37 reference. The filtered gene-barcode matrices from CellRanger were used for further analysis with Rstudio (version 4.3.2) and the Seurat package (version 5.0.2). Looking only at the sample hashing the data was first normalized using NormalizeData (normalization.method = “CLR”) and then demultiplex using HTODemux (positive.quantile = 0.99) and only cells classified as Singlet were used for the downstream analysis.

For the analysis of gene expression, genes identified in more than 3 cells and cells containing over 200 detected genes were included. To filter out low-quality cells, the following criteria were applied: 1) fewer than 6,000 detected genes per cell, 2) fewer than 25,000 molecules identified per cell, and 3) greater than 5% of Unique Molecular Identifiers (UMIs) originating from mitochondrial DNA. Following the removal of these low-quality cells, the SCTransform function was used for normalization of the gene expression matrices. Cell cycle effects were mitigated by regressing out S phase and G2/M phase scores during the data scaling process. Following this filtration, we had a total of 5,606 cells; 3,494 cells for donor BC349 and 2,112 cells for donor BC357 were included in further analyses. Additionally, donor-specific variations (BCs) were minimized through integration using the Harmony function from Seurat.

We previously reported the comparison between the unexpanded, free peptide, and Ag-IL2/21 scaffold expansion strategies (11). However, in this study, we have expanded our dataset by incorporating the two Ag-Neo2/15 scaffolds (Neo-3x and Neo-12x) into the single cell data. This addition allows for a comprehensive comparison of the Ag-Neo2/15 scaffolds with the previously used expansion strategies.

### Gene set enrichment analysis

The differential expressed genes, found between either Ag-IL2/21 and Ag-Neo2/15 (Neo-3x) or Ag-Neo2/15 (Neo-12x) scaffolds, were ranked by their average log2 fold changes and a gene set enrichment analysis (GSEA) was performed with the gseGO function from clusterProfiler (version 4.10.1) (18), using only biological process (ont=‘BP’) with a minimum gene set above 10 (minGSSize = 10), and with a p-value cutoff below 0.05 (pvalueCutoff = 0.05). The resulting pathways were further filtered to have a qvalue < 0.1 and a GeneRatio > 0.5, and the enrichment score for each of these pathways are shown in **Supplementary Figure 7**. GSEA plots for selected enriched pathways were visualized using the gseaplot2 function from the enrichplot package in R.

### Gene enrichment scores

To evaluate the effect of multiple genes on a single cell level, we used the AddModuleScore function from Seurat to calculate enrichment scores for different gene sets, namely the cytotoxic, dysfunctional, and proliferation scores. The cytotoxic and dysfunctional score was calculated using the top30 cytotoxic and dysfunctional genes identified by Li et al. (13). Here the dysfunctional gene set consists of LAG3, HAVCR2, PDCD1, PTMS, FAM3C, IFNG, AKAP5, CD7, PHLDA1, ENTPD1, SNAP47, TNS3, CXCL13, RDH10, DGKH, KIR2DL4, LYST, MIR155HG, RAB27A, CSF1, CTLA4, TNFRSF9, CD27, CCL3, ITGAE, PAG1, TNFRSF18, GALNT1, GBP2, and MYO7A, and the cytotoxic gene set includes IL7R, GNLY, FGFBP2, CX3CR1, FCGR3A, S1PR5, PLAC8, FGR, C1orf21, SPON2, CD300A, TGFBR3, PLEK, STK38, S1PR1, EFHD2, KLRF1, C1orf162, SORL1, FCRL6, TRDC, EMP3, CCND3, KLRG1, BIN2, SELL, KLRB1, SAMD3, and ARL4C.

The proliferation score was calculated using a gene set with the following genes, CCND3, CDK2, CDK4, CDK6, MKI67, E2F1, E2F2, PCNA, MCM2, MCM3, MCM4, MCM5, MCM6, MCM7, TOP2A, MYC, BCL2 and BCL2L1.

### T-cell receptor analysis

For the TCR analysis, we used only cells with at least one productive TCR α-chain and/or one productive TCR β-chain, selecting only the chain with the highest UMI count and read count if multiple chains were found within a cell. The clonotype ID was then manually annotated based on unique CDR3 sequences from the α- and β-chain. The CDR3 α and β sequences of the top four TCR clones were aligned using the imgt_align_junctions function from the cdr3tools library (https://github.com/caparks2/cdr3tools). The multiple sequence alignment and logo plot was performed using the ggmsa function from the ggmsa library (22).

### Phenotype analysis

The cell surface protein analysis was performed using immunophenotyping antibodies from the TotalSeq™-C library from 10XGenomics, and the data was normalized and scaled using the NormalizeData and ScaleData functions from Seurat.

### Differential expression analysis

To identify differentially expressed genes and phenotype markers across all experimental expansion strategies, Seurat’s *FindAllMarkers* function was applied to both data types, respectively, using parameters *logfc.threshold = 0* and *min.pct = 0.75.* The resulting markers were filtered to include only those with an adjusted p-value < 0.001 and an average log2 fold change > 2. These selected markers were ranked within each condition and visualized using heatmaps generated with the ComplexHeatmap package.

To find differential expressed genes or phenotype makers between either Ag-IL2/21 and Ag-Neo2/15 (Neo-3x) or Ag-Neo2/15 (Neo-12x) scaffolds, the FindMarkers function from Seurat was used, with the logfc.threshold = 0 and min.pct = 0.75. Differential expressed genes or phenotype makers were shown using volcano plots generated with ggplot2.

## Data Availability

The original contributions presented in the study are publicly available. The single-cell sequencing dataset analyzed in this study have been deposited in the NCBI Gene Expression Omnibus (GEO) repository under accession number GSE329498 (https://www.ncbi.nlm.nih.gov/geo/query/acc.cgi?acc=GSE329498). Additional data supporting the conclusions of this article are included in the article and its **Supplementary Material**.
