# Supplementary material for: Antigen-scaffolds loaded with hyper-stable neoleukin-2/15 expand antigen-specific T cells with a favorable phenotype for adoptive cell therapy
Source: Front Immunol. 2026 Jun 11;17:1804678. doi: 10.3389/fimmu.2026.1804678 (PMC13294229; doi:10.3389/fimmu.2026.1804678)
Supplement: Supplementary file 1 [file DataSheet1.pdf]

Supplementary figures

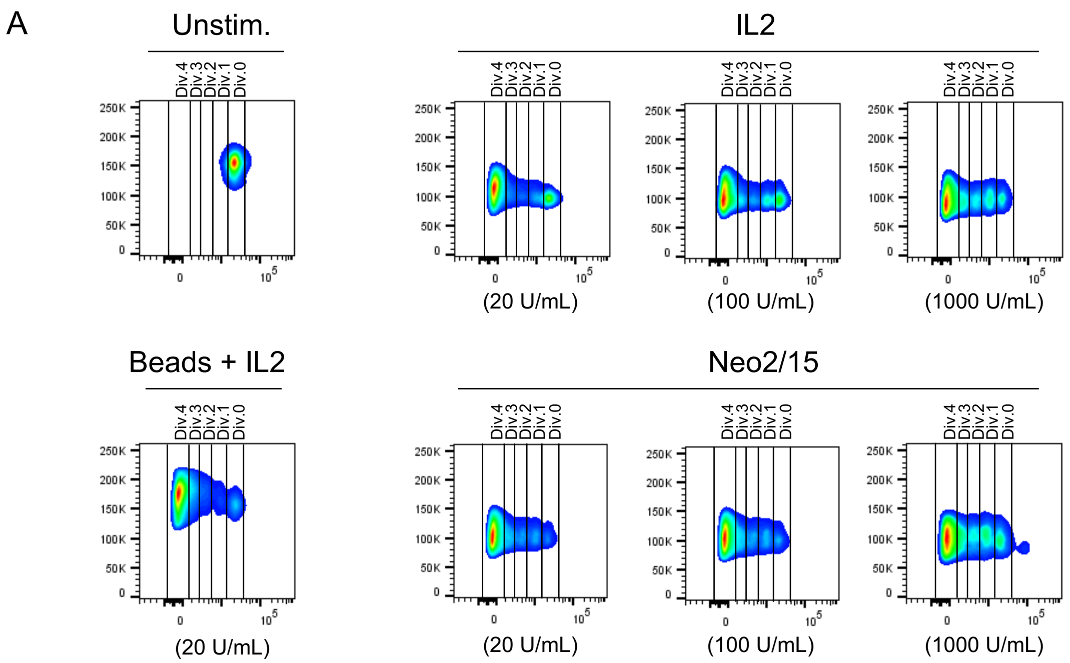

B

Nr. of divisions	Unstim.	Beads + IL2	IL2 20U/mL	IL2 100U/mL	IL2 1000U/mL	Neo2 /15 20U/mL	Neo2 /15 100U/mL	Neo2 /15 1000U/mL
Div.0	93.1 %	4.8%	14.2%	11.4%	6.3%	3.1%	3.8%	8.6%
Div.1	4.0%	1.7%	4.1%	6.1%	7.4%	3.9%	4.4%	8.0%
Div.2	0.4%	2.8%	3.2%	4.7%	7.4%	4.7%	5.2%	8.2%
Div.3	0.1%	9.9%	6.3%	9.2%	10.6%	8.6%	10.0%	13.3%
Div.4	0.2%	78.5%	70.7%	67.1%	66.7%	78.3%	75.0%	58.6%

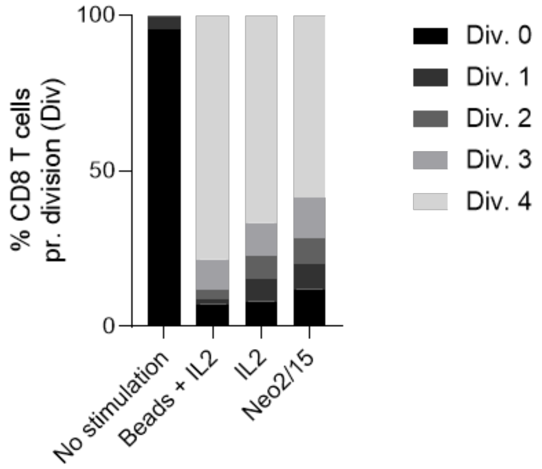

Supplementary Figure 1: T cells stimulated with Neo2/15-avi and IL2 proliferate to a similar level. A) Flow plots showing the division of CD8^{+} T cells stimulated with molar ratios of IL2 or Neo2/15-avi as seen by dilution of CFSE cell trace dye. Unstimulated T cells were used a negative control and T cells stimulated with Dynabeads and Il2 as positive control. B) Proportion of CD8^{+} T cells in each division under the indicated stimulation conditions. C) Barograph showing the number of divisions between expansion conditions for cells stimulated with 1000U/ml cytokine (n=2 donors).

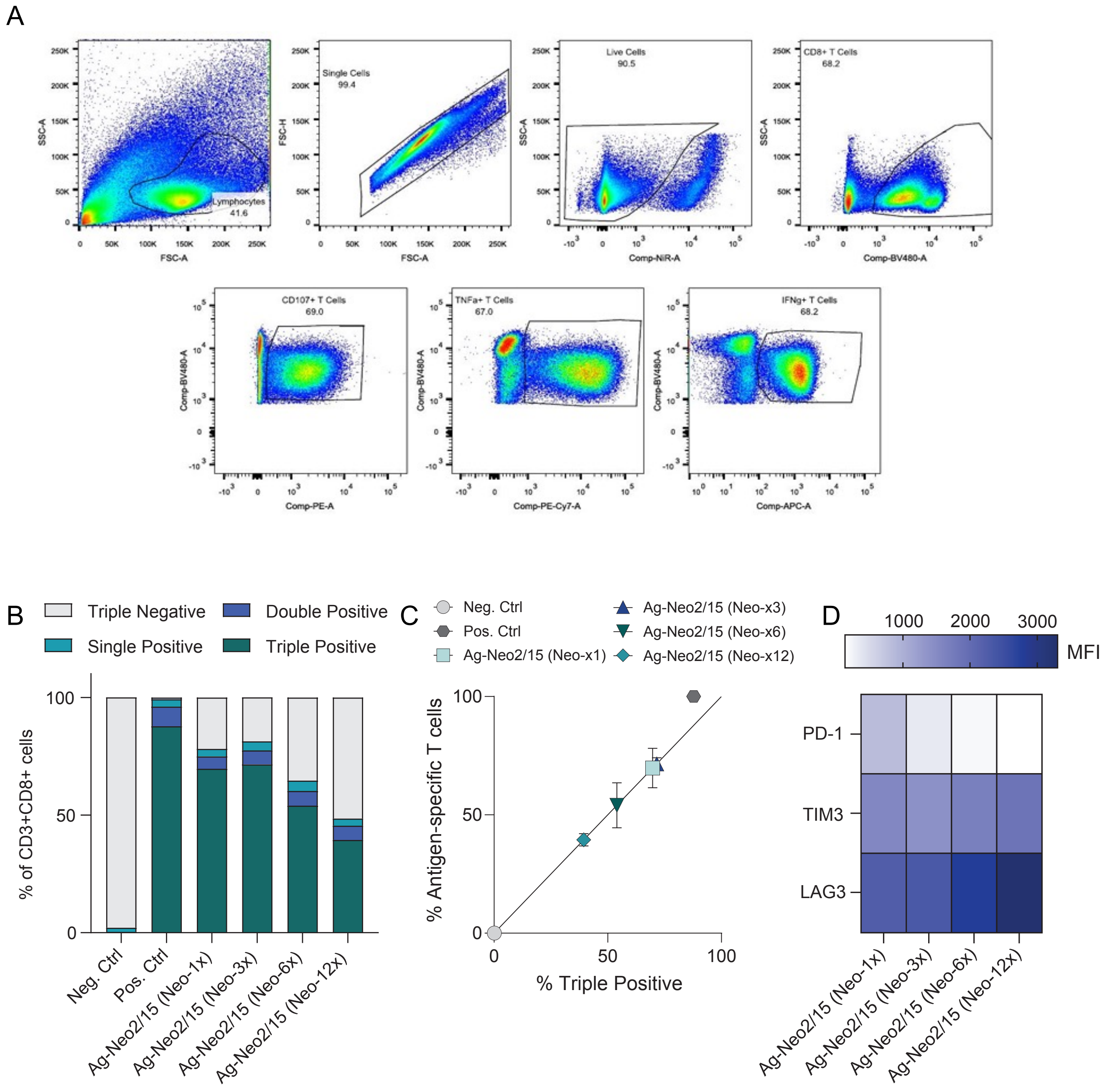

Supplementary Figure 2: Polyfunctional and exhaustion profiles of T cells expanded with Ag-Neo2/15 scaffolds. A) The gating strategy for identifying CD107a, TNFα, IFNγ+ CD8+ T cells in cultures expanded with Ag-scaffolds and co-cultivated with target cells. B) The percentage of cells in culture expressing one, two, or three cytokines (CD107a, TNFα, IFNγ) as determined by intracellular cytokine staining (ICS). Specifically, the negative control consists of target cells not pulsed with antigenic peptide, while the positive control corresponds to lymphocyte activation cocktail (LAC) stimulation. C) The relationship between % Triple positive cytokine T cells and the % of antigen-specific cells in culture. D) The mean fluorescence intensity of exhaustion markers PD-1, TIM3, and LAG3 on the total CD8+ T cells expanded with Ag-Neo2/15 scaffolds determined by flow cytometry. Data is shown for T cells from 1 donor expanded in duplicate (n=2).

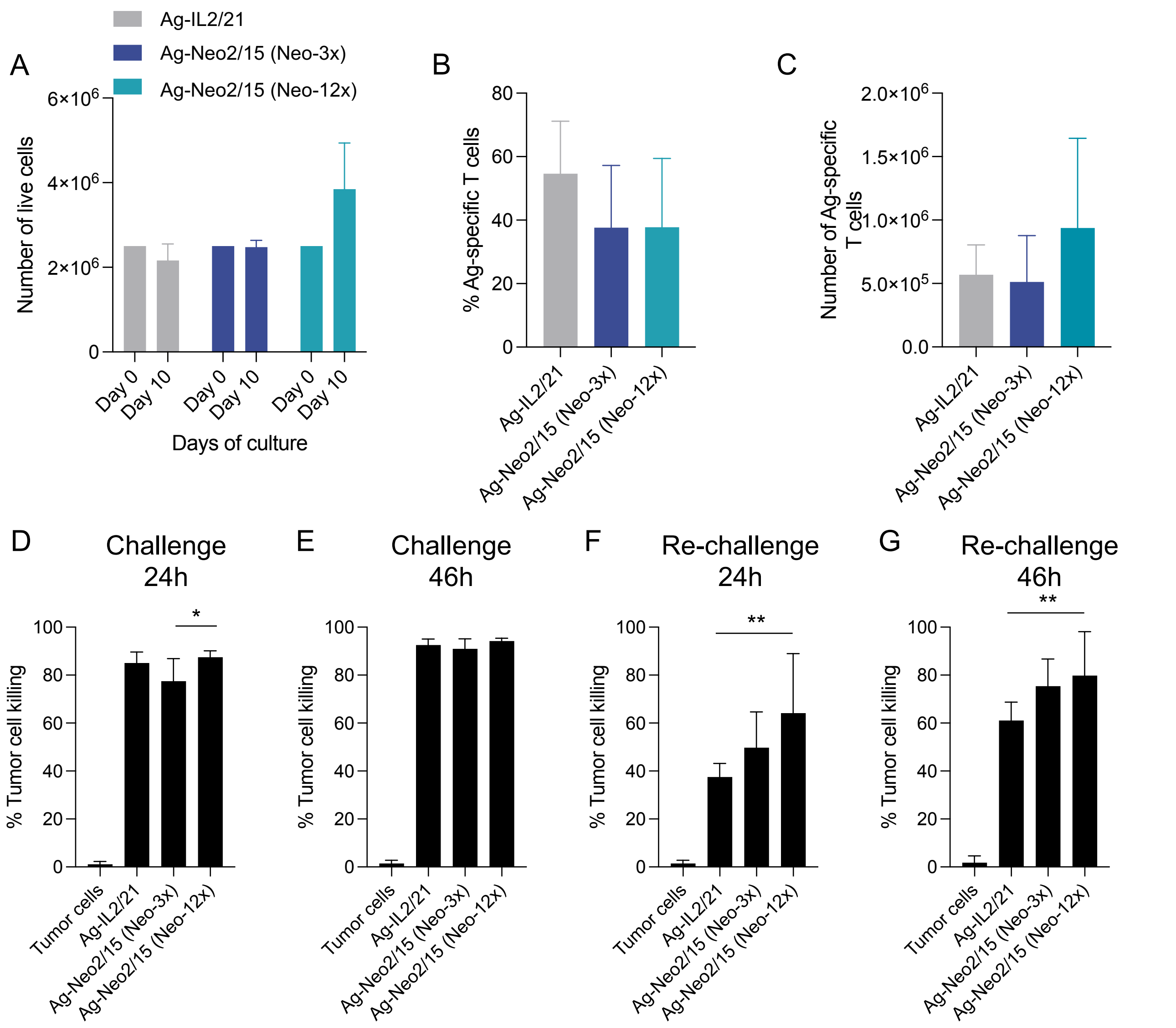

Supplementary Figure 3: The expansion kinetics and cytotoxic capacity of CMV specific T cells expanded with Ag-Reg or Ag-Neo2/15 scaffolds.
A) The number of live cells in cultures expanded with Ag-scaffolds for 10 days. B) % of antigen-specific T cells in cultures at day 0 and 10 as determined by tetramer-staining and flow cytometry. C) The number of antigen-specific T cells at day 0 and 10 of culture. The maximum target cell killing at D) 24h and E) 46h after initial co-cultivation with Ag-scaffold expanded T cells. The maximum cell killing at F) 24h and G) 46h after T cells are re-challenged with target cells. The statistical test shown between the different expansion strategies were compared using a one-way ANOVA test (D – F) and , where p values > 0.05 were considered statistically non significant and represented as ns, whereas p ≤ 0.05, p < 0.01, p < 0.001 and p < 0.0001 is represented as *, **, *** and ****, respectively. Data is shown for T cells expanded from 3 donors (n=3).

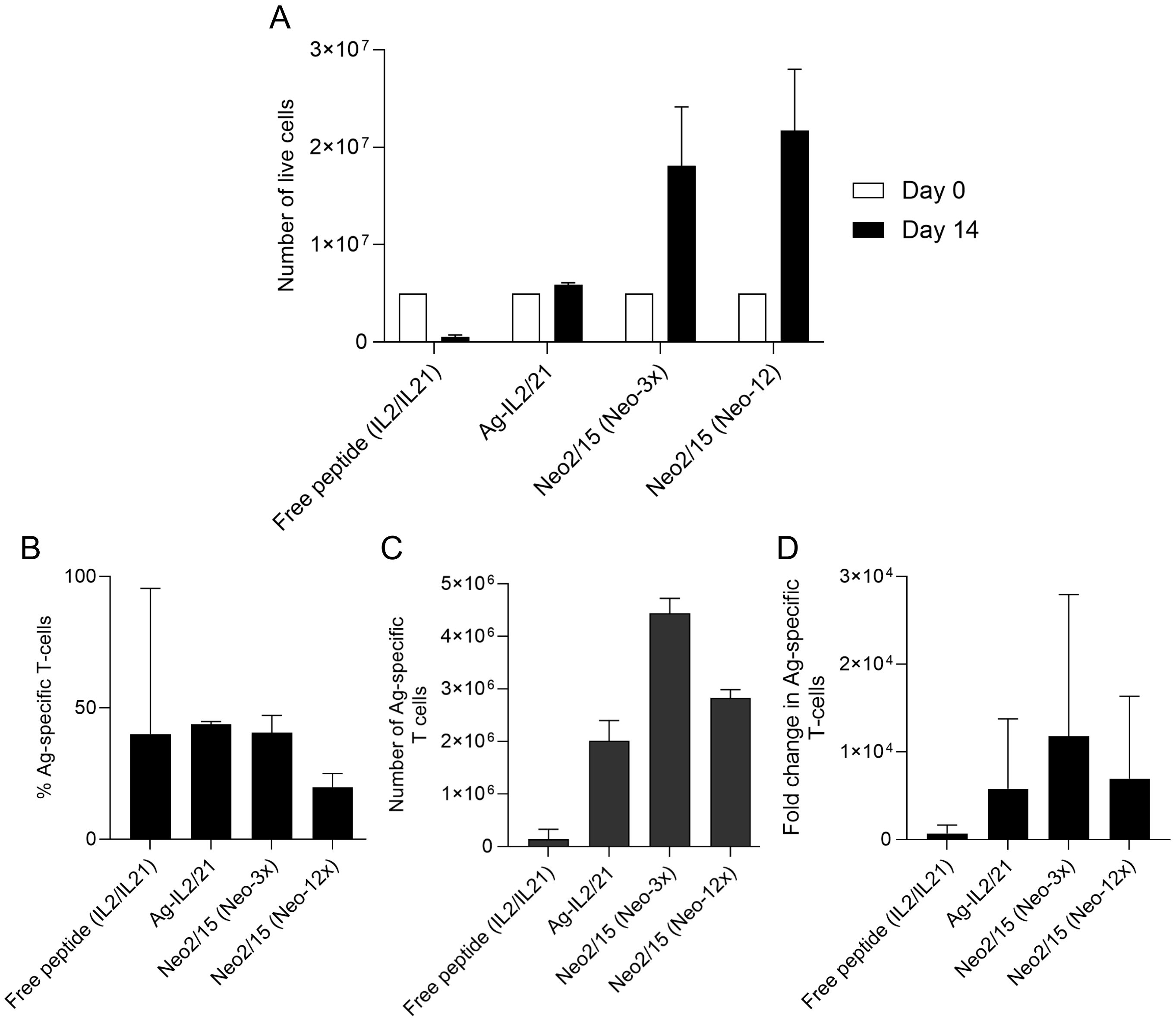

Supplementary Figure 4: Expansion kinetics of T cells expanded with Ag-scaffolds prior to single cell analysis.
A) Number of live cells after a 14 day expansion period with Ag-scaffolds or free-peptide. B) The percentage of antigen-specific CD8+ T cells after 14 days of expansion as determined by antigen-tetramers and flow cytometry. C) The number of antigen-specific cells after 14 days of expansion in cultures expanded with Ag-scaffolds or free peptide. D) The fold expansion of antigen-specific cells after 14 days of expansion with either Ag-scaffolds or free-peptide. The statistical test shown between the different expansion strategies were compared using a one-way ANOVA test (B – D) and , where p values > 0.05 were considered statistically non significant. Data is shown for T cells expanded from 2 donors (n=2).

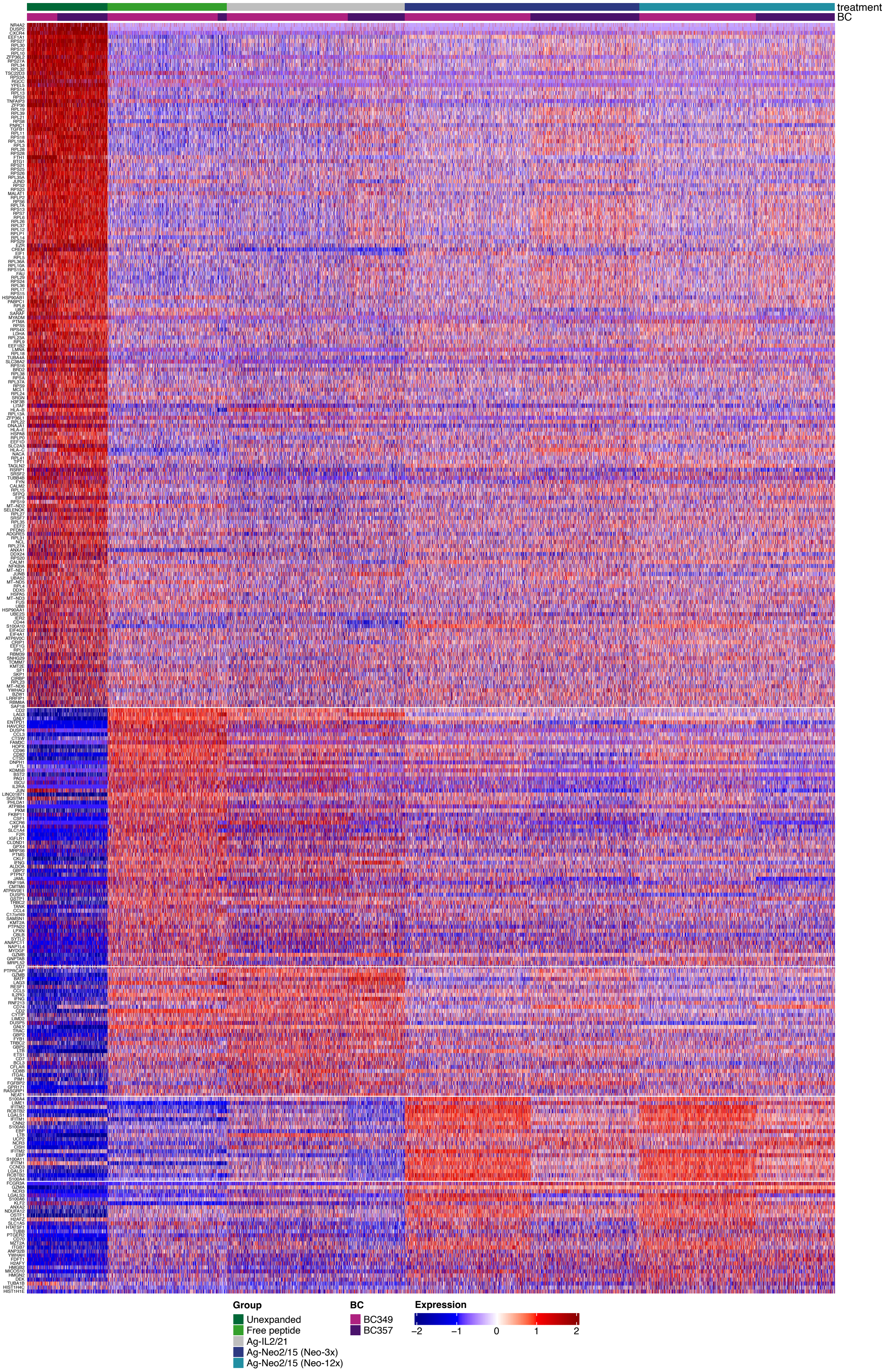
treatment
BC
Group
Unexpanded
Free peptide
Ag-IL2/21
Ag-Neo2/15 (Neo-3x)
Ag-Neo2/15 (Neo-12x)
BC
BC349
BC357
Expression
−2
−1
0
1
2

Supplementary Figure 5: Heatmap of most differentially expressed genes for each expansion strategy. Heatmap from the single-cell analysis from the RNA panel, showing the most differentially expressed genes for each expansion strategy, clustering the cells for each healthy donor within each expansion strategy. Data is shown for T cells expanded from 2 donors (n=2).

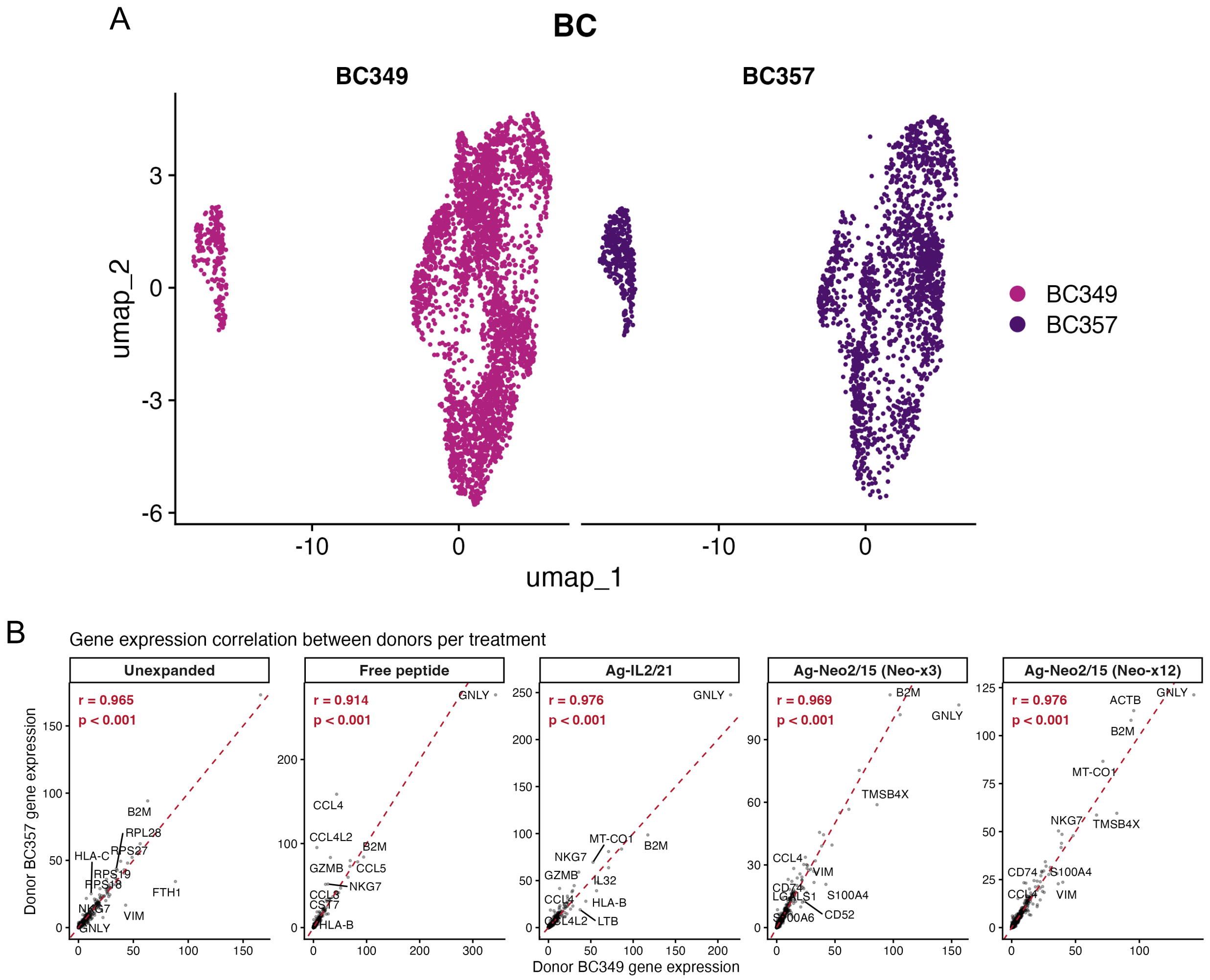

Supplementary Figure 6: Donor concordance across expansion strategies.
A) UMAP of single-cell transcriptomes, split by donor. B) Scatter plot of average gene expression per expansion strategy, comparing donor BC349 and donor BC357. Pearson correlation coefficients for each strategy are indicated. Data is shown for T cells expanded from 2 donors (n=2).

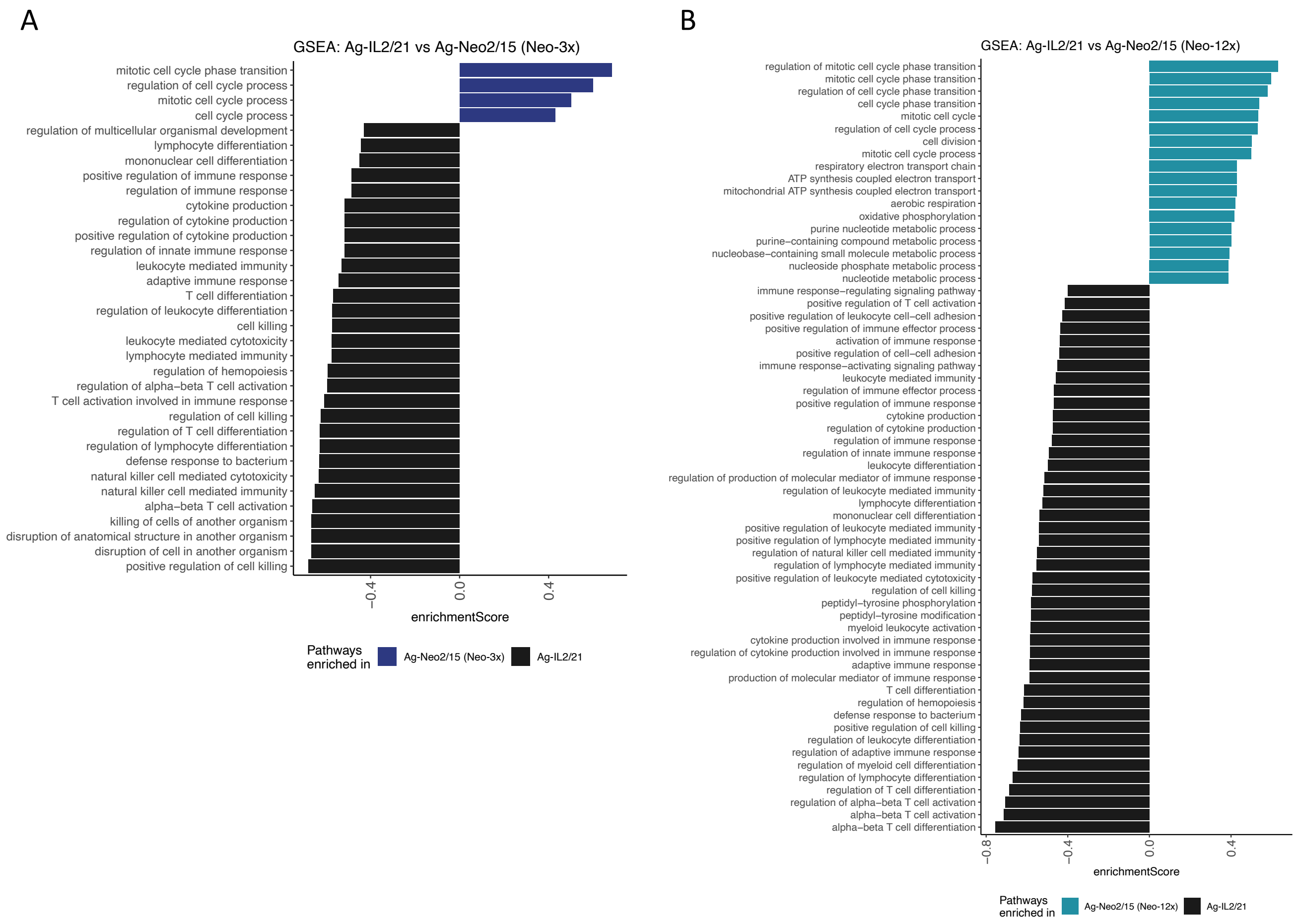

Supplementary Figure 7: Summary of all the biological pathways found in the GSEA.
Enrichment scores for the biological pathways, determined by the gene set enrichment analysis (GSEA) when comparing A) Ag-IL2/21 and Ag-Neo2/15 (Neo-3x) and when comparing B) Ag-IL2/21 and Ag-Neo2/15 (Neo-12x). Data is shown for T cells expanded from 2 donors (n=2).

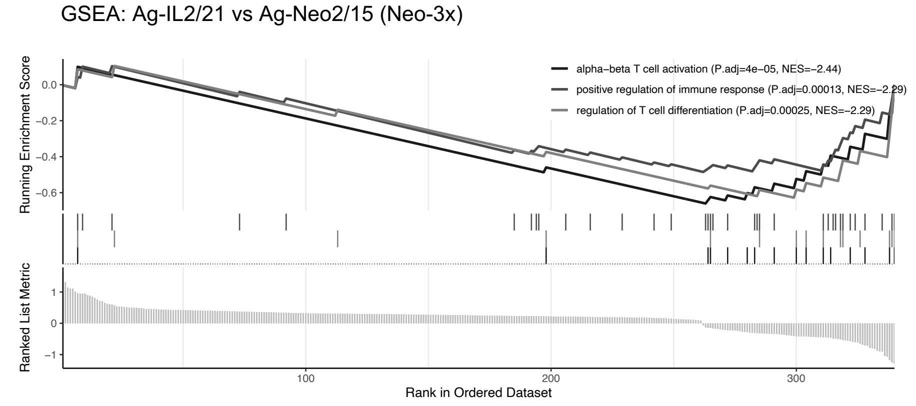

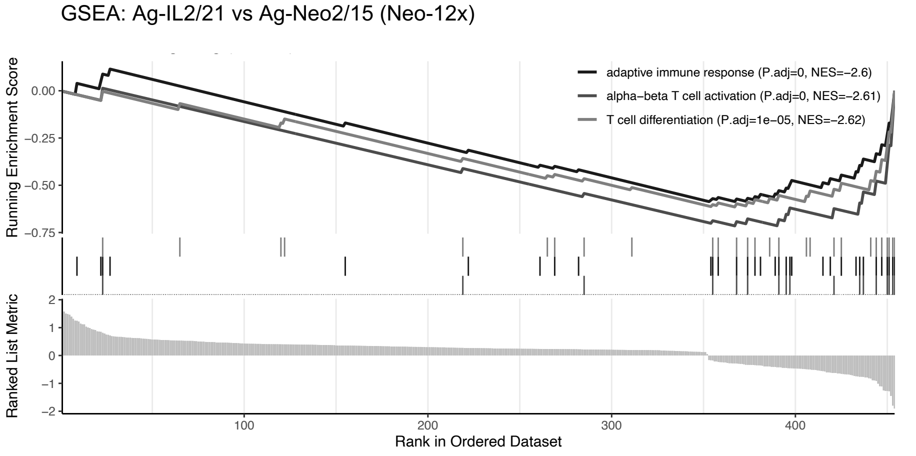

Supplementary Figure 8: Selected biological pathways found in the GSEA enriched in Ag-IL2/21. Selected biological pathways determined by the gene set enrichment analysis when comparing Ag-IL2/21 vs Ag-Neo2/15 (Neo-3x) (top) and when comparing Ag-IL2/21 vs Ag-Neo2/15 (Neo-12x) (bottom). Data is shown for T cells expanded from 2 donors (n=2).

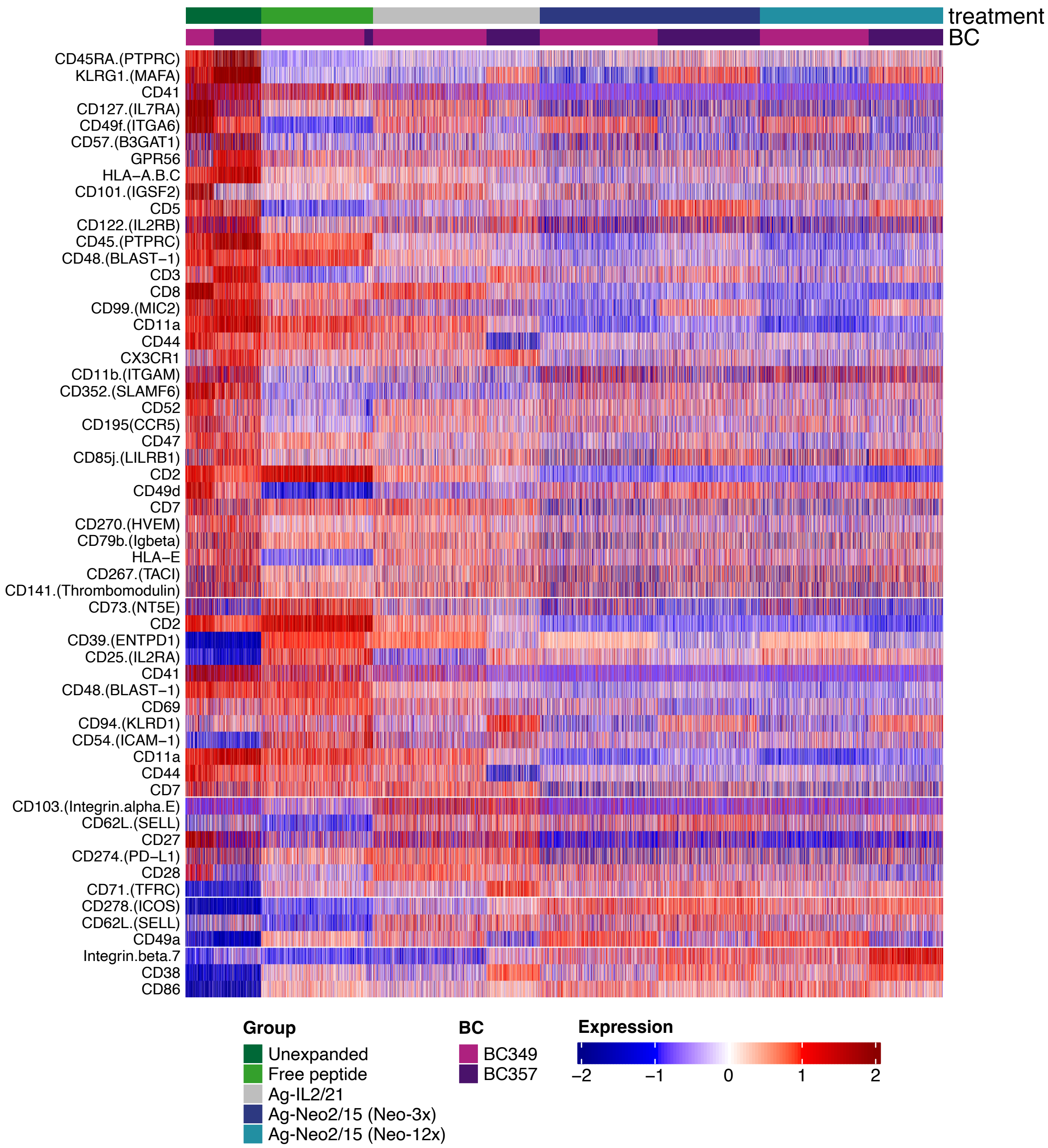

Supplementary Figure 9: Most differentially expressed surface markers from the phenotype panel. Heatmap from the single-cell analysis phenotype panel, showing the most differentially expressed surface markers for each expansion strategy, clustering the cells for each healthy donor within each expansion strategy. Data is shown for T cells expanded from 2 donors (n=2).

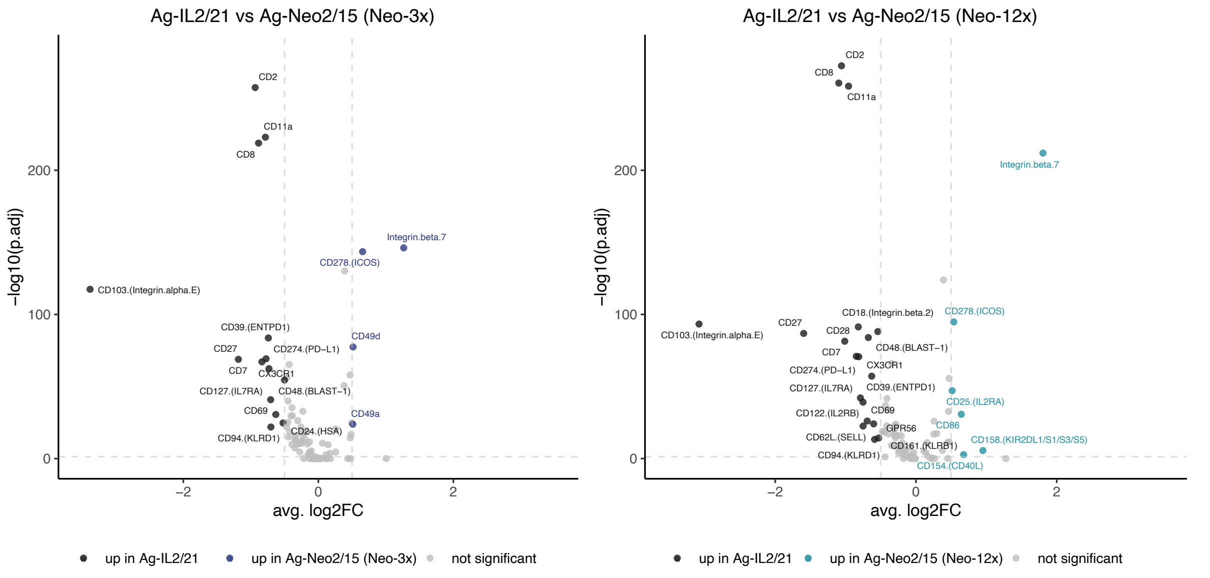

Supplementary Figure 10: Most differentially expressed surface markers from the phenotype panel. Volcano plot showing key differentially expressed surface markers from the phenotype panel, between the Ag-IL2/21 and Ag-Neo2/15 (Neo-3x) (left panel) and between the Ag-IL2/21 and Ag-Neo2/15 (Neo-12x) (right panel). Data is shown for T cells expanded from 2 donors (n=2).

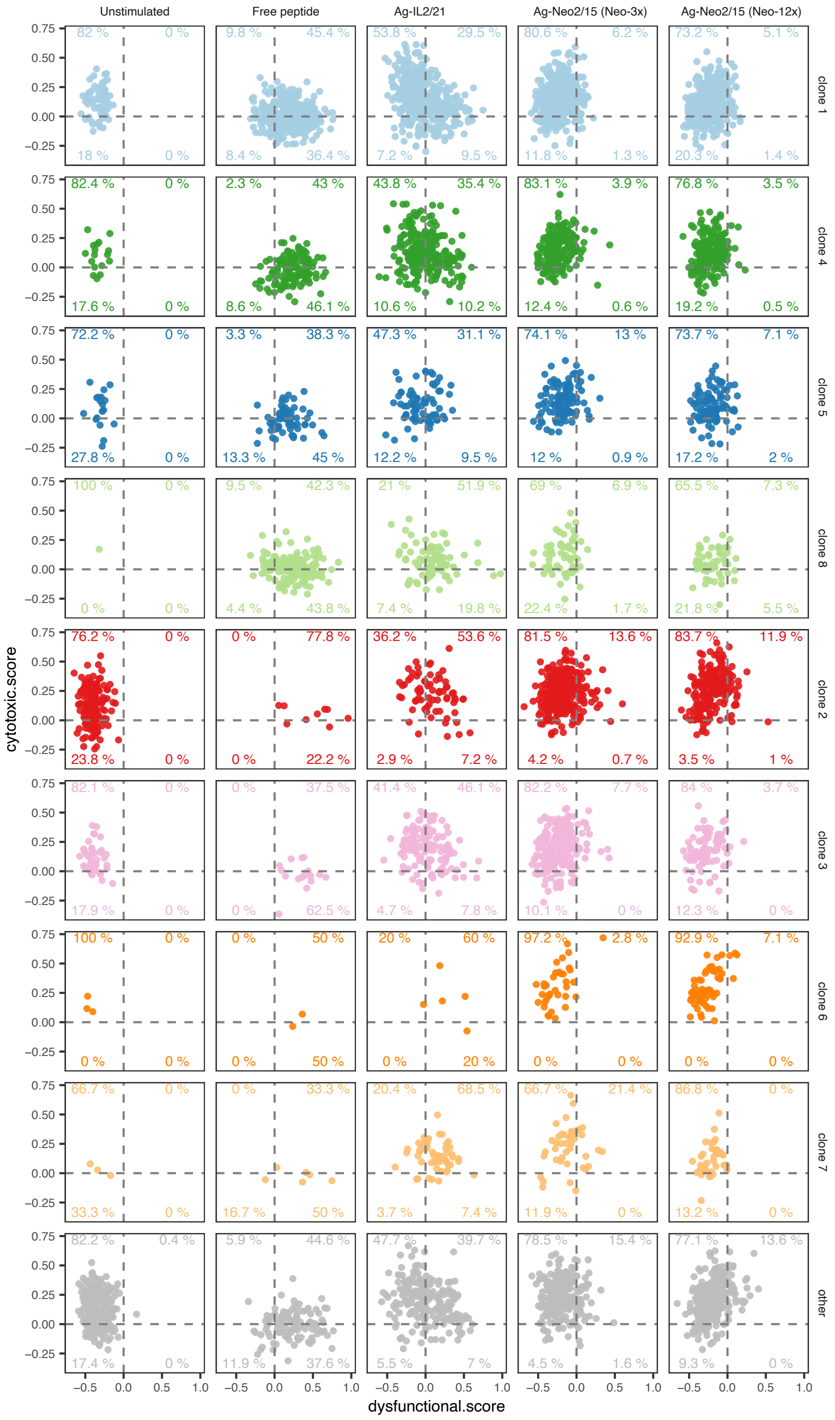

Supplementary Figure 11: Relationship between the cytotoxic and dysfunctional score for the top four TCR clones for each donor.

Quadrant scatter plots showing the relationship between the cytotoxic and dysfunctional score for each the top four TCR clones within each donor. Each scatter plot is divided into four quadrants. Quadrants with cyt+dys+ show cells where both scores are above the zero, cyt-dys- means that both scores are below zero, cyt+dys- represents cells where the cytotoxic score is above zero and the dysfunctional score is below zero and the cyt-dys+ are cells where the cytotoxic score is below zero and the dysfunctional score is above zero. Data is shown for T cells expanded from 2 donors (n=2).
